# Pneumococcal Vaccination Utilization Among Hispanic Long-Term Colorectal Cancer Survivors: Cross-Sectional Assessment of Claims

**DOI:** 10.2196/12603

**Published:** 2019-05-13

**Authors:** Ryan J Moran, Jill Waalen, James Murphy, Vinit Nalawade, Melody Schiaffino

**Affiliations:** 1 Department of Family Medicine and Public Health University of California San Diego La Jolla, CA United States; 2 School of Public Health San Diego State University San Diego, CA United States; 3 3Scripps Translational Research Institute San Diego, CA United States; 4 Department of Radiation Medicine and Applied Sciences University of California San Diego La Jolla, CA United States; 5 Institute for Behavioral and Community Health San Diego State University San Diego, CA United States

**Keywords:** Hispanic Americans, cancer survivors, medicare, preventive medicine, pneumococcal vaccines

## Abstract

**Background:**

Colorectal cancer (CRC) is the second leading cancer-related cause of death in the United States. However, survivorship has been increasing. Both cancer survivors and underserved populations experience unique health-related challenges and disparities that may exist among long-term CRC survivors as it relates to routine preventive care, specifically pneumococcal (PNM) vaccination.

**Objective:**

The aim of this study was to explore the relationship between long-term CRC survival and the receipt of PNM vaccine among Hispanic Medicare recipients compared with non-Hispanic populations.

**Methods:**

This study is a cross-sectional analysis of the Surveillance, Epidemiology, and End Results (SEER)-Medicare claims data examining ethnic differences in the receipt of PNM vaccination among long-term CRC survivors. Multivariable logistic regression models considered Hispanic ethnicity while controlling for sociodemographic characteristics, comorbidity score, age, tumor stage, and SEER registry.

**Results:**

Our sample revealed 32,501 long-term CRC survivors, and 1509 identified as Hispanic (4.64%) based on an established SEER algorithm. In total, 16,252 CRC survivors, or 50.00% of our sample, received a PNM vaccination. We found that Hispanic CRC survivors had 10% decreased odds of having received a PNM vaccine compared with non-Hispanic survivors (*P*=.03).

**Conclusions:**

Disparities likely exist in the utilization of PNM vaccination among long-term CRC survivors. Among Medicare beneficiaries, the use of claims data regarding PNM vaccination highlights the relatively poor utilization of guideline-directed preventive care.

## Introduction

### Background

Colorectal cancer (CRC) persists as the third most common cancer in the United States among both men and women [1-3]. Although it remains the second leading cause of death from cancers that affect both men and women [2], there has been a significant decrease in CRC-related mortality in the last 40 years [1,2]. Lifestyle modification, screening for early detection, and advances in treatment for individuals with CRC have led to an increase in 5-year survival rates from 50% to 66%. In the last decade—where data are available—the annual predicted rate of change for CRC mortality has decreased by 2.9% [1,3]. As a result of this, the number of long-term survivors has been steadily increasing. At present, an estimated 1.4 million CRC survivors are living in the United States. Of these, over 85% are aged older than 60 years [4,5].

Among cancer survivors, studies have found conflicting findings regarding routine preventive care utilization and health care utilization compared with noncancer survivors. For example, survivors were more likely to report pneumococcal (PNM) and influenza vaccination, blood pressure monitoring, cholesterol measurement, bone mineral scans, and lower endoscopy via the *Medicare Current Beneficiary* Survey [6]. However, a study using Surveillance, Epidemiology, and End Results *(* *SEER)-Medicare* data suggests decreased influenza vaccination, cholesterol screening, cervical cancer screening, and bone density screening [7]. Another study using a combined metric for overall appropriate care utilization from the *SEER-Medicare* database found overall decreased care in CRC survivors compared with noncancer cases [8,6].

Hispanics/Latinos represent the biggest minority racial or ethnic group in the United States, currently estimated at 17.6% of the population. By 2060, this group is predicted to represent over 28% of the population [9]. Although Hispanic/Latino individuals are less likely to be diagnosed with CRC and have a lower mortality rate than non-Hispanic whites [1,2], healthy Hispanic/Latinos are also less likely to receive routine preventive care [10,11] such as CRC screening. Furthermore, evidence suggests that under-represented minority populations who survive cancer may be less likely to receive other essential routine preventive care such as influenza vaccination [6], including long-term survivors of CRC [12]. Additional evidence from the *National Health Interview Survey* suggests that Hispanic/Latino cancer survivors are less likely to utilize health care services compared with non-Hispanic whites for those aged 65 years and younger, though this did not appear to be the case for survivors aged 65 years and older. Some explanations include less time in the health care system, lower socioeconomic capacity to access services, and barriers such as out-of-pocket expenses [13].

### Objectives

Medicare represents the most significant public insurer in the United States, with over 55 million beneficiaries and more than 37 million of these on a traditional fee-for-service model [13]. Over 2 million beneficiaries are Hispanic/Latinos enrolled in a traditional Medicare plan [14],^ ^and Medicare Part B has been providing coverage for the PNM vaccine since 1981 with no out-of-pocket expenses [15]. Since 1984, vaccination has been advised for all adults aged 65 years and older [16]. In the general population, evidence suggests that Hispanic adults aged 65 years and older are less likely to have received a PNM vaccine and are less likely to know that it is advisable [10,17]. However, evidence evaluating how Hispanic or Latino long-term survivors of CRC fare with regard to PNM vaccination recommendations appears limited [6,7].

We sought to explore the relationship between long-term CRC survival and the receipt of PNM vaccine among Hispanic/Latino Medicare recipients compared with non-Hispanic/Latino populations, given that the majority of new CRC cases occur in those aged 65 years and older [2].

## Methods

### Study Design

Our study is a retrospective, cross-sectional analysis of patients diagnosed with CRC identified within an existing dataset of the SEER program linked with the *Center for Medicare and Medicaid Services* (CMS) administrative claims database for the years 2000 to 2011 (N=318,675).

### Population

SEER-Medicare data references information for patients with Medicare diagnosed with cancer, with a tumor registered at one of the National Cancer Institute’s (NCI) 20 registries across the United States. These registries were then matched to CMS’s administrative claim record enrollment file and had a 94% match. The dataset covers SEER diagnostic information for up to 10 diagnosed cancer occurrences for cases recorded in the SEER cancer registries [18]. Socioeconomic information derived from the US Census has been included for the years 1990 to 2000.

The Medicare data contain outpatient claims for diagnoses or specific procedures as defined by the International Classification of Diseases, Ninth Revision, Clinical Modification (ICD-9-CM) codes and the Healthcare Common Procedure Coding System (HCPCS) codes along with their dates of service.

### Eligibility

The cases were those patients aged 66 years and older who were diagnosed with a first-primary tumor located in the colon or rectum, CRC, at any stage. We defined CRC as those cancers using the site-recode ICD-0-3/World Health Organization 2008 codes: 21041, 21042, 21043, 21044, 21045, 21046, 21047, 21048, 21049, 21051, and 21052. Tumors had to be histologically confirmed and first-primaries. Survivors were included if they lived at least 5 years beyond the time of diagnosis.

**Figure 1:**
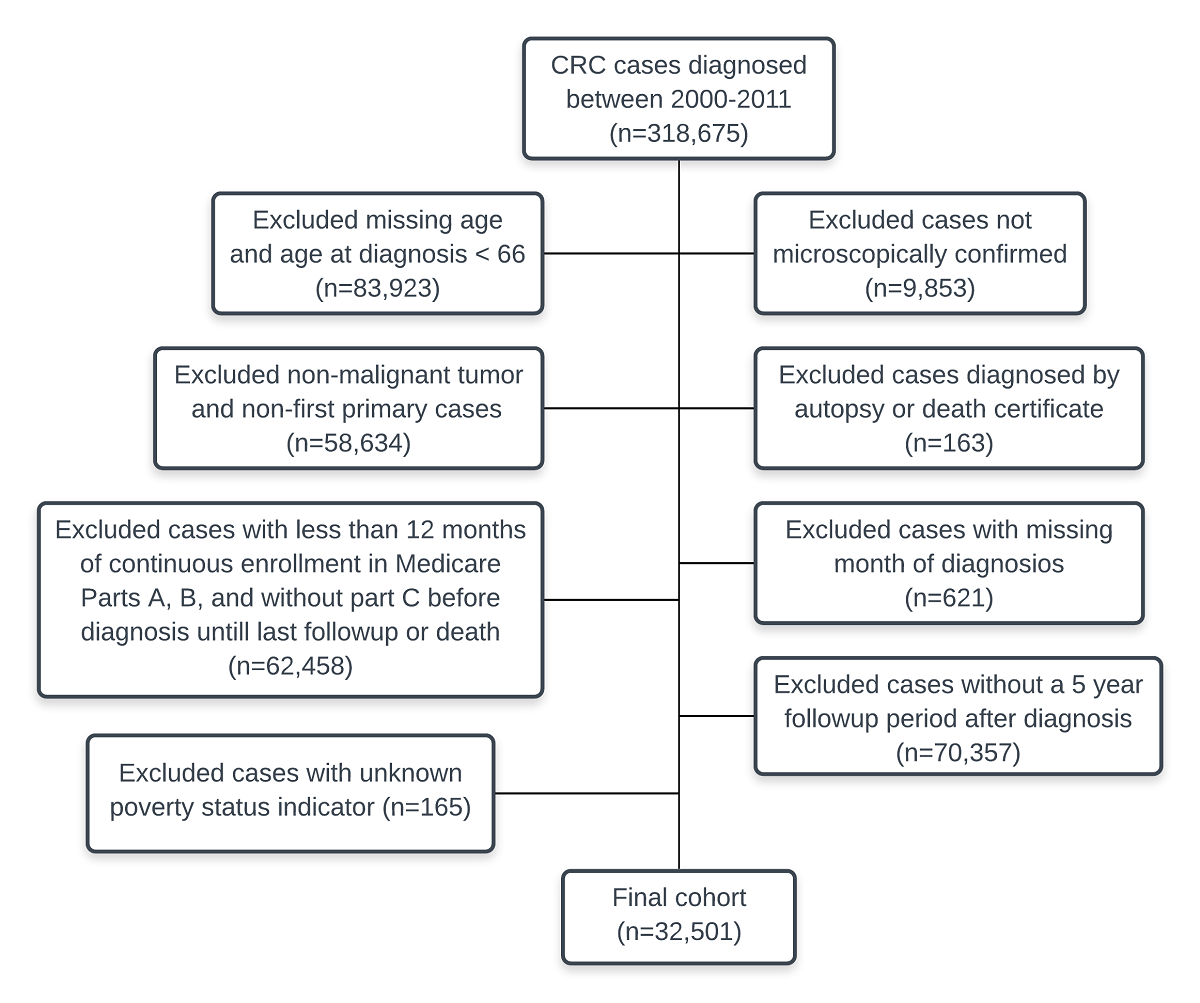
Flow diagram defining cases of long-term colorectal cancer (CRC) survivors.

We excluded patients with missing ages or those who had been diagnosed by autopsy or death certificate. Patients with a missing month at diagnosis and patients with other primary cancers predating CRC diagnosis were also excluded. Cases with missing data such as the federal poverty category *unknown* were also excluded.

We selected patients who had continuous coverage of both Medicare Parts A and B but without Part C coverage from at least 1 year before diagnosis until the last follow-up or death to allow for 1 complete year of claims data before diagnosis to calculate a comorbidity score. To assure subjects were long-term survivors, we narrowed our study sample to those with at least 5 years of follow-up after cancer diagnosis. Time was calculated as the months from SEER-database entry to the point of death or end of follow-up (ending December 31, 2013), as has been described in a previously published cross-sectional approach that accounted for censorship [19]. The final SEER-Medicare dataset for analysis included 32,501 observations (Figure 1).

### Exposure

SEER data were also used to extract some variables at diagnosis including patient age, sex, marital status, and race/ethnicity per the North American Association of Cancer Registries (NAACR) Hispanic Identification Algorithm (NHIA) Derived Hispanic Origin. NHIA has an estimated sensitivity of 84% and a positive predictive value of over 94% for identifying those of Hispanic descent [20]. As we sought to explore vaccination status by those of ethnic identity, regardless of racial background, SEER data on race/ethnicity were used to create a dichotomous Hispanic/non-Hispanic variable.

### Outcome

To assess for utilization of preventive services, specifically, PNM vaccination, claims data from Medicare identified the following ambulatory-based encounters wherever vaccination ICD-9-CM codes were involved: V03.82 and V06.6; using the Current Procedural Terminology (CPT) codes: 90670 and 90732; and the HCPCS code: G0009. As CRC survivors had a varied age of diagnosis, all data regarding immunization claims were included in our analysis regardless if this was before or after the time of diagnosis.

### Covariates

Age at diagnosis was categorized into 5-year increments from 65 to 85 years. Although age at diagnosis and the date of PNM immunization claim varied chronologically, patients were included in the analysis as long as they reported a claim for an immunization within the study period. For instances of multiple vaccinations, we used the earliest CPT/HCPCS claim and used the ICD-9 claim wherever a CPT/HCPCS claim date was absent. We also extracted the stage of disease (histology confirmed) given this likely greatly influenced visits to a health care setting, extracted the SEER registry site given the regional variation noted in preventive service utilization [21,22], and extracted a poverty indicator: the percent of the population who reside in the census-designated region at or below the federal poverty level (FPL). In addition, we included characteristics regarding the place of residence as either a metropolitan area or any other nonmetro area. Medicare date of death for the linked data from Medicare claims was used to confirm SEER month and year of death and the first day of the month when the Medicare date was not available (0.003% of the sample). Patient health status was assessed using the Charlson comorbidity scoring algorithm, an index to evaluate patient comorbidities with a SEER-Medicare–provided macro that included 10 comorbidities noted between the ages of 65 and 66 years: moderate/severe liver disease, cerebrovascular disease, peripheral vascular disease, renal disease, paralysis (hemiplegia or paraplegia), dementia, mild liver disease, congestive heart failure, chronic obstructive pulmonary disease, diabetes with complications, and diabetes [23-26]. The ICD-9 values for comorbidities were referenced from the comorbidity index found at the NCI [27,28]. Registries are geographically defined by SEER and correspond to different regions and cities to be representative of the American population. Of the original 20 SEER registry categories, 17 were available and recategorized if they had less than 50 Hispanic individuals, which was found to equate to less than 2% of the corresponding registry. All the remaining registries in our database were combined into 1 category.

To account for the variation of additional time since the 5-year survival cut-point of time, we included months after 5 years from diagnosis in our model (variable not reported in tables, available upon request).

### Data Management and Statistical Analyses

Data exploration was conducted on all variables of interest to assess distribution, cut-points, and group differences. Descriptive statistics for all categorical variables and means for continuous variables are reported. Variables of interest were compared based on our primary outcome variable, namely any receipt of PNM vaccine by the ethnic group using a chi-square analysis to consider the goodness-of-fit for modeling. Multivariable logistic regression models were fitted to independent variables of interest to our outcome. With a subanalysis using only those patients that did utilize a vaccine (n=16,171), we evaluated whether or not the place of service influenced receipt of vaccines among Hispanic/Latino versus non-Hispanic CRC survivors. We compared place of service by vaccine utilization across Hispanic/Latino versus non-Hispanic groups using a chi-square analysis to explore group differences. All data management and analyses were performed using SAS version 9.4 (SAS Institute Inc). The University of California-San Diego Human Research Protection Institutional Review Board Program approved this study, and data were utilized with agreement and approval from SEER-Medicare.

## Results

### Sample Characteristics

Our sample included 32,501 long-term CRC survivors, with a mean age of diagnosis of 76.6 years. The majority were diagnosed at a local stage (57.5%), were women (56.95%), were married (53.76%), and resided in a nonrural setting (87.79%). Over 50% of CRC survivors were between the ages of 66 and 75 years, and nearly two-thirds had an identified modified Charlson comorbidity score of 0 (67%). Over half (55%) resided in census-designated tracts where 10% or less of the population earned at or below 100% of the FPL. Overall, 50% (n=16,252) of CRC survivors received a PNM vaccination. Of these, approximately 1509 long-term CRC survivors were identified as Hispanic/Latino, compared with 30,992 who were identified as non-Hispanic (Table 1).

Statistically significant differences for both receipt of PNM vaccine and between Hispanic and non-Hispanic groups for gender, modified Charlson Comorbidity Score, categorized age of diagnosis, percent at or below the FPL, urban versus nonurban setting, marital status, and SEER registry location were noted. There was a statistically significant difference between the histology stage at diagnosis for PNM vaccine recipients but not by racial/ethnic group (Table 2).

**Table 1:** Characteristics of long-term colorectal cancer survivors in a Surveillance, Epidemiology, and End Results-Medicare database (2000 to 2011; N=32,501).

SEER^a^-Medicare characteristic and value		n (%)
**Hispanic/Latino (SEER-North American Association of Cancer Registries Hispanic Identification Algorithm identity)**		
	Hispanic/Latino	1509 (4.64)
	Non-Hispanic	30,992 (95.36)
**Received a pneumococcal vaccine**	
	Yes	16,252 (50.00)
	No	16,249 (50.00)
**Age at diagnosis (years)**	
	66-69	5367 (16.51)
	70-74	8207 (25.25)
	75-79	8283 (25.49)
	80-84	6407 (19.71)
	85+	4237 (13.04)%
**Gender**	
	Male	13993 43.05%
	Female	18508 (56.95)
**Urban status**	
	Urban/Metro	28,533 (87.79)
	Rural	3968 (12.21)
**Census tract federal poverty level (%)**	
	0 to <5	9464 (29.12)
	5 to <10	9161 (28.19)
	10 to <20	8795 (27.06)
	>20	5081 (15.63)
**Number of comorbidities (Charlson Index)**	
	0	21807 (67.10)
	1	7339 (22.58)
	2	2372 (7.30)
	3 or more	983 (3.0)
**Marital status**	
	Divorced	1800 (5.54)
	Married	17473 (53.76)
	Other	10,987 (33.81)
	Single	2241 (6.90)
**Tumor stage at diagnosis**	
	Localized	18,689 (57.50)
	Regional	12,297 (37.84)
	Distant	870 (2.7)
	Unstaged	645 (1.98)
**SEER registry region**	
	San Francisco	1119 (3.44)
	Connecticut	2261 (6.96)
	San Jose	721 (2.2)
	Los Angeles	2120 (6.52)
	Greater California	5124 (15.77)
	New Jersey	5620 (17.29)
	New Mexico	744 (2.3)
	Other^b^	14792 (45.51)

^a^SEER: Surveillance, Epidemiology, and End Results.

^b^Combined if less than 50 in the registry identified as Hispanic*.*

**Table 2:** Characteristics of Hispanic/Latino and non-Hispanic colorectal cancer survivors who received a pneumococcal vaccination.

Characteristics		Received PNM^a^ vaccine, n (%)			Did not receive PNM vaccine, n (%)		
		Hispanic (n=727, 4.5%)	Non-Hispanic (n=15525, 95.53%)	*P* value	Hispanic (n=782, 4.8%)	Non-Hispanic (n=15,467; 95.19%)	*P* value
**Age at diagnosis (years)**	
	66-69	158 (21.7)	2465 (15.88)	<.001	157 (20.1)	2587 (16.73)	<.001
	70-74	210 (28.9)	4094 (26.37)	<.001	226 (28.9)	3677 (23.77)	<.001
	75-79	195 (26.8)	4027 (25.94)	<.001	202 (25.8)	3859 (24.95)	<.001
	80-84	106 (14.6)	3069 (19.77)	<.001	126 (16.1)	3106 (20.08)	<.001
	85+	58 (8)	1870 (12.05)	<.001	71 (9)	2238 (14.47)	<.001
**Gender**	
	Male	360 (49.5)	6541 (42.13)	<.001	394 (50.4)	6698 (43.31)	<.001
	Female	367 (50.5)	8984 (57.87)	<.001	388 (49.6)	8769 (56.69)	<.001
**Urban status**	
	Urban	678 (93.3)	13879 (89.40)	<.001	735 (94)	13241 (85.61)	<0001
	Rural	49 (7)	1646 (10.60)	<.001	47 (6)	2226 (14.39)	<.001
**Federal Poverty Level (%)**			
	0 to <5	107 (14.7)	4956 (31.92)	<.001	125 (16.0)	4276 (27.65)	<.001
	5 to <10	146 (20.1)	4471 (28.80)	<.001	159 (20.3)	4385 (28.35)	<.001
	10 to <20	251 (34.5)	4002 (25.78)	<.001	269 (34.4)	4273 (27.63)	<.001
	20 to 100	223 (30.7)	2096 (13.50)	<.001	229 (29.3)	2533 (16.38)	<.001
**Charlson Comorbidity Index**			
	0	400 (55.0)	9947 (64.07)	<.001	510 (65.2)	10950 (70.8)	.006
	1	200 (27.5)	3811 (24.55)	<.001	181 (23.2)	3147 (20.35)	.006
	2	81 (11)	1261 (8.12)	<.001	65 (8)	965 (6.2)	.006
	3 or more	46 (6)	506 (3.3)	<.001	26 (3)	405 (2.6)	.006
**Marital status**			
	Divorced	46 (6)	812 (5.2)	.03	54 (7)	888 (5.7)	.04
	Married	403 (55.4)	8606 (55.43)	.03	403 (51.5)	8061 (52.12)	.04
	Other	215 (29.6)	5095 (32.82)	.03	253 (32.4)	5424 (35.07)	.04
	Single	63 (9)	1012 (6.52)	.03	72 (9)	1094 (7.07)	.04
**Tumor stage at diagnosis**			
	Localized	416 (57.2)	9088 (58.54)	.92	406 (51.9)	8779 (56.76)	.03
	Regional	279 (38.4)	5781 (37.24)	.92	335 (42.8)	5902 (38.16)	.03
	Distant	19 (3)	388 (2.5)	.92	19 (2)	444 (2.9)	.03
	Unstaged	13 (2)	268 (1.7)	.92	22 (3)	342 (2.2)	.03
**Surveillance, Epidemiology, and End Results registry region**							
	San Francisco	41 (6)	516 (3.3)	<.001	35 (4)	527 (3.4)	<.001
	Connecticut	29 (4)	1151 (7.41)	<.001	24 (3)	1057 (6.83)	<.001
	San Jose	39 (5)	364 (2.3)	<.001	31 (4)	287 (1.9)	<.001
	Los Angeles	104 (14.3)	975 (6.3)	<.001	125 (15.98)	916 (5.9)	<.001
	Greater California	230 (31.6)	2360 (15.20)	<.001	278 (35.6)	2256 (14.59)	<.001
	New Jersey	113 (15.5)	2800 (18.04)	<.001	126 (16.1)	2581 (16.69)	<.001
	New Mexico	104 (14.3)	288 (1.9)	<.001	89 (11)	263 (1.7)	<.001
	Other	67 (9)	7071 (45.55)	<.001	74 (9)	7580 (49.01)	<.001

^a^PNM: pneumococcal.

### Pneumococcal Vaccination Among Hispanic and Non-Hispanic Colorectal Cancer Survivors

The results from our adjusted multivariable model indicated 10% decreased odds of vaccination for Hispanic CRC survivors versus non-Hispanics, which is marginally statistically significant (adjusted odds ratio [AOR] 0.889, 95% CI 0.798 to 0.99; *P*=.033; Table 3).

Characteristics associated with increased odds of PNM vaccine receipt included being between ages 70 and 74 years and 75 and 79 years compared with those between aged 65 and 69 years, an increasing modified Charlson comorbidity index, being married, and being female (*P*<.05 for all). As the percent of a population in a census tract at the FPL increased, receipt of PNM vaccine odds decreased (less than 5% at FPL with AOR 1.31, 95% CI 1.213 to 1.408; *P*=<−.01; between 10% to 19.9% at FPL AOR 1.108, 95% CI 1.033 to 1.189; *P*=.004). A subgroup comparison (Table 4) of long-term CRC survivors by Hispanic identify revealed differences in place of receipt of PNM vaccination (*P*=.030).

**Table 3:** Adjusted odds of receiving a pneumococcal vaccine among long-term colorectal cancer survivors (Surveillance, Epidemiology, and End Results-Medicare 2000 to 2011; N=32,501).

Characteristic and category		Receipt of pneumococcal vaccination		
		Adjusted odds ratio	95% CI	*P* value
**Hispanic/Latino (SEER^a^-North American Association of Cancer Registries Hispanic Identification Algorithm identity)**				
	No	Ref^b^	—^c^	—
	Yes	0.888	0.798-0.990	.032
**Age at diagnosis (years)**	
	66-69	Ref	—	—
	70-74	1.14	1.063-1.222	<.001
	75-79	1.055	0.984-1.132	.13
	80-84	0.991	0.920-1.068	.82
	>85	0.848	0.780-0.923	<.001
**Charlson Comorbidity Score**	
	0	Ref	—	—
	1	1.344	1.274-1.418	<.001
	2	1.47	1.348-1.602	<.001
	3 or more	1.437	1.262-1.636	<.001
**Marital status**	
	Single	Ref	—	—
	Married	1.148	1.049-1.255	.003
	Divorced	0.967	0.853-1.096	.60
	Other	1.004	0.914-1.101	.94
**Urban status**	
	Urban or Metro	1.298	1.206-1.397	<.001
	Rural	Ref	—	
**Census tract federal poverty level (%)**	
	0 to <5	1.307	1.213-1.408	<.001
	5 to <10	1.181	1.100-1.267	<.001
	10 to <20	1.108	1.033-1.189	.004
	20 to 100	Ref	—	—
**Gender**	
	Male	Ref	—	—
	Female	1.139	1.086-1.194	<.001
**Tumor stage at diagnosis**	
	Local	Ref	—	—
	Regional	0.941	0.898-0.985	.009
	Distant	0.856	0.746-0.982	.03
	Unstaged	0.779	0.664-0.914	.002
**SEER registry region**	
	San Francisco	0.964	0.851-1.086	.56
	Connecticut	1.020	0.930-1.120	.67
	San Jose	1.220	1.046-1.421	.01
	Los Angeles	1.036	0.943-1.138	.46
	Greater California	1.039	0.972-1.110	.26
	New Jersey	1.000	0.936-1.069	>.99
	New Mexico	1.312	1.127-1.526	<.001
	All Others	Ref	—	—

^a^SEER: Surveillance, Epidemiology, and End Results.

^b^Serves as reference group, adjusted odds ratio not applicable.

^c^Not applicable.

**Table 4:** Places of service utilized by Hispanic and non-Hispanic colorectal cancer survivors who received their first pneumococcal vaccination in a Surveillance, Epidemiology, and End Results-Medicare cohort (N=16,171; *P*=.030).

Vaccine site of administration^a^	Total, N (%)	Non-Hispanic, n (%)	Hispanic, n (%)
Physician’s office	12,012 (74.28)	11,482 (74.33)	530 (73.3)
Hospital	1499 (9.27)	1415(9.2)	84 (11.6)
Home/Home Health Agency, Nursing Facility or Custodial Care	749 (4.6)	720 (9.2)	29 (4)
Specialty centers	207 (1.3)	192 (1.2)	15 (2)
Community Care Centers	1704 (10.54)	1639 (10.61)	65 (9)

^a^Place of service was excluded if unlisted.

## Discussion

### Principal Findings

To our knowledge, this is the first study evaluating the utilization of preventive services by long-term survivors of CRC, utilizing claims data. A recent study assessed preventive medical care in long-term survivors of CRC utilizing similar methods but looked only at Asian Pacific Islanders [19] and another study looked at preventive services in long-term cancer survivors using survey data, finding lower rates in Hispanic and African Americans [29]. We noted a marginally significant difference in PNM vaccination utilization among those who identified as Hispanic versus non-Hispanic using a robust, large dataset and controlling for confounding variables. Clinically, although small, this difference is important given the anticipated growth in Hispanic Americans.

Importantly, we also found that claims data show that only approximately 50% of Medicare beneficiaries, overall, received at least 1 PNM vaccine. This estimate lags not only behind the *Healthy People 2020* goal of 90% utilization but also behind current estimates of PNM vaccination in populations aged over 65 years [30,25]. Furthermore, this number is markedly lower than results from the most recent Medicare Beneficiary Survey (2013) where 69.1% of respondents answered *yes* to the question “Have you ever had a shot for pneumonia?” [30]; however, it is more consistent with rates found for all beneficiaries with Medicare claims through part A and B [31].

### Strengths and Limitations

One strength of our study was limiting our subject population to long-term CRC survivors—a group that presumptively has had precedented contact with the medical community; furthermore, Medicare claims data and the study limitation to those aged 65 years and older should help mitigate medical need and payment ability, given our primary outcome should have no direct expense to individuals. Our study further controlled for socioeconomic status and morbidity burden. Identification of those who received a vaccine, either by CPT or ICD-9 classification provided a more robust ability to signify access to health systems, as previously cited concerns in the literature include lack of insurance, access, or knowledge of vaccine recommendation.

Our study had limitations. First, as we utilized any receipt of PNM vaccine at any time via Medicare claims data, many individuals may have opted to defer Medicare B enrollment until after the age of 65 years but may have received the vaccine with an alternative payer. Second, although these data were captured so long as Medicare was the payer, the data fail to establish the temporal relation of survivorship before preventive service updates, and thus our study fails to inform on the importance of this preventive service in this population. Third, claims data do not permit for understanding those who may have been offered vaccination and declined to receive it. Fourth, our data rely on claims from Medicare; although this is likely comprehensive, it may fail to include some individuals with alternative payment mechanisms, including cash. Finally, our comorbidity score was calculated for all patients based on their claims from 1 year before diagnosis until 1 day before diagnosis. It is possible that individuals had different health protectors that may have influenced their vaccine status.

### Conclusions

Future studies should consider better metrics to distinguish vulnerable populations, specifically Hispanic-Americans. Claims data permit for well-powered studies to evaluate the impact and meaning of certain preventive services, for example, rates of hospitalization for disseminated PNM disease, versus those without this service. Cost-effectiveness data are also lacking in these groups, which could help health care professionals, administrators, and policy-makers reach better decisions to allocate resources. Finally, adherence to guideline-directed care and overutilization of certain services should be explored, given some individuals in our study received more than 3 vaccines.

Previous studies citing health care provider recommendations as important mediators to vaccination utilization are limited in that they rely on survey data. Survey data can introduce recall bias, which may allow adverse selection for those who have received services, inappropriately denoting increased utilization. Those who have foregone this preventive service may have done so for a number of reasons, including lack of a provider visit, lack of recall of getting this vaccine or misunderstanding of specifics (eg, influenza vaccination being mistaken for PNM vaccine), lack of knowledge of vaccine significance or importance, or actual lack of occurrence. In addition, we found numerous beneficiaries receiving more than 3 vaccines (not reported) highlight concern for overutilization of resources: although guidelines and best practices support certain high-risk individuals who receive additional vaccinations pending clinical circumstances, it is permissive that other vaccines were administered because of a lack of communication and a segregated health care system.

Providers, health care managers, and informational system specialists should all carry some concern and responsibility; utilization of preventive services (PNM vaccine) is relatively low in our study population, and system-wide recognition is needed to rectify this. Although shared decision making and patient-centered care remain important, directed initiatives and resource allocation may help improve these findings and increase the utilization of evidence-based routine preventive care.

## Abbreviations

AOR: adjusted odds ratio
CMS: Center for Medicare and Medicaid Services
CRC: colorectal cancer
CPT: Current Procedural Terminology
FPL: federal poverty level
HCPCS: Healthcare Common Procedure Coding System
ICD-9-CM: International Classification of Diseases, Ninth Revision, Clinical Modification
NAACR: North American Association of Cancer Registries
NCI: National Cancer Institute
NHIA: NAACR Hispanic Identification Algorithm
PNM: pneumococcal
SEER: Surveillance, Epidemiology, and End Results
